# Global spread of dengue virus types: mapping the 70 year history

**DOI:** 10.1016/j.tim.2013.12.011

**Published:** 2014-03

**Authors:** Jane P. Messina, Oliver J. Brady, Thomas W. Scott, Chenting Zou, David M. Pigott, Kirsten A. Duda, Samir Bhatt, Leah Katzelnick, Rosalind E. Howes, Katherine E. Battle, Cameron P. Simmons, Simon I. Hay

**Affiliations:** 1 Spatial Ecology and Epidemiology Group, Department of Zoology, University of Oxford, South Parks Road, Oxford OX1 3PS, UK; 2 Department of Entomology, University of California Davis, Davis, California 95616, USA; 3 Fogarty International Center, National Institutes of Health, Bethesda, Maryland 20892, USA; 4 Department of Zoology, University of Cambridge, Cambridge, CB2 3EJ, UK; 5 Oxford University Clinical Research Unit, Hospital for Tropical Diseases, Ho Chi Minh City, Vietnam; 6 Centre for Tropical Medicine, University of Oxford, Churchill Hospital, Oxford OX3 7LJ, UK; 7 Nossal Institute of Global Health, University of Melbourne, Parkville, Victoria, Australia

**Keywords:** dengue, serotypes

## Abstract

Since the first isolation of dengue virus (DENV) in 1943, four types have been identified. Global phenomena such as urbanization and international travel are key factors in facilitating the spread of dengue. Documenting the type-specific record of DENV spread has important implications for understanding patterns in dengue hyperendemicity and disease severity as well as vaccine design and deployment strategies. Existing studies have examined the spread of DENV types at regional or local scales, or described phylogeographic relationships within a single type. Here we summarize the global distribution of confirmed instances of each DENV type from 1943 to 2013 in a series of global maps. These show the worldwide expansion of the types, the expansion of disease hyperendemicity, and the establishment of an increasingly important infectious disease of global public health significance.

## Highlights

• The geography of type-specific global DENV circulation has not been well described.
• We map the global distribution and co-circulation of each DENV type from 1943 to 2013.
• Detection of all types has expanded worldwide together with growing hyperendemicity.
• There remains a dearth of type-specific information in many parts of the world.

## Early spread and typing of DENV

DENV are members of the *Flavivirus* genus (see Glossary), related to other medically important arboviruses such as yellow fever and Japanese encephalitis viruses. There are four phylogenetically and antigenically distinct dengue viruses (DENV1–4), and although infection with one type confers long-term immunity, it is to that type only and not to the other three [1]. The ancestor of these viruses has been postulated to have emerged about 1 000 years ago in an infectious cycle involving non-human primates and mosquitoes, with transmission to humans having occurred independently for all four virus types only a few hundred years ago 2, 3. Although outbreaks of disease clinically consistent with dengue have been reported for centuries, it was not until 1943 in Japan and 1945 in Hawaii that the first two dengue viruses were isolated (named DENV1 and DENV2, respectively) [4]. At this point, epidemics of dengue illness were being reported across the region spanning from India to the Pacific Islands. In the latter half of the 20th century, DENV transmission followed the spread of its principal mosquito vector, *Aedes aegypti* [5], and was likely accelerated by urbanization and globalization 6, 7. The collapse of the *Ae. aegypti* eradication campaign in the Americas in the 1970s was also important in marking the beginning of transport of Asian dengue viruses to the Americas, followed by the rapid re-introduction of the principal mosquito vector throughout both continents [8].

## A need for type-specific global maps

Spatial patterns in concurrent and/or sequential circulation of DENV1–4 should be considered along with virus and host genetics as potentially important population-level risk factors for severe dengue illness 9, 10 because secondary infection with a heterologous DENV type may increase the probability of severe disease 11, 12, 13, 14. Despite this, no study has systematically reviewed all documented spatially explicit evidence of the global spread of the four DENV types since the first isolation in 1943. Rather, the majority of existing studies have focused on the evolution of individual DENV types at regional or local scales 15, 16, 17, 18, 19, 20. Global descriptions of type-specific DENV distribution are few in number, lacking spatial and temporal precision, and are presented in a non-systematic manner [8].

Reported cases do not comprise the entire range of each DENV type at any given time, meaning that a lack of reporting for a specific type at any time or place does not indicate its certain absence. This is due to spatial variability in several factors, namely in the degree of sampling, proportion of infections having been typed, reliability of typing methods, and finally reporting of these types. That said, the use of more advanced typing methods has expanded significantly across the globe since their development, and a thorough description of confirmed presences of each DENV type is needed if we are to gain a better understanding of the global dispersal of the four viruses and track changes moving forward. Here we provide this baseline depiction, also highlighting those geographic areas lacking in information about the specific DENV type(s) responsible for dengue occurrence.

Brady *et al.* [21] recently outlined the definitive extents of dengue presence globally, and their work was followed by that of Bhatt *et al.* [22] which generated high spatial-resolution (5 km × 5 km) estimates of contemporary global dengue risk and burden in 2010. Our efforts here provide further insight into the global distribution of dengue by reviewing the individual DENV types responsible for reported occurrence throughout the past seven decades. Our aim is to complement phylogenetic and disease occurrence analyses by presenting the sub-national distribution of reported confirmed instances of human infection with each DENV type globally from 1943 to 2013. We provide a more spatially and temporally detailed picture of the spread of each individual DENV type than was previously available, also presenting contemporary maps of the number of DENV types ever reported in an area to elucidate global patterns in their co-circulation and establishment of hyperendemicity.

## Compiling a global database of DENV type reporting

We compiled an extensive database by extracting DENV type and locational information from published literature and case reports spanning the period 1943–2013. In brief, searches were conducted in PubMed (http://www.ncbi.nlm/pubmed) using the terms ‘dengue’ and ‘serotype’ or ‘type,’ and all pseudonyms were automatically included using the Medical Subject Headings (MeSH) terminology. No language restrictions were placed on these searches; however, only those citations with a full title and abstract were retrieved. In-house language skills allowed processing of all English, French, Portuguese, and Spanish articles. We were unable to extract information from a small number of Turkish, Polish, Hebrew, Italian, German, and Chinese articles. ProMED reports (http://www.promedmail.org) were also searched using the terms ‘dengue’ and ‘serotype’ or ‘type’, and all DENV type data that could be linked to a location were extracted, resulting in a search of 1 912 unique articles or reports. DENV type data from national Ministry of Health websites were included where available, resulting in 294 additional sources of information. Finally, DENV envelope protein gene sequences were extracted from GenBank (http://www.ncbi.nlm.nih.gov/genbank), providing another 1 070 sources for a total of 3 276 sources from which type and geographical coordinate information was ultimately extracted. References for these sources are available upon request.

Geo-positioning was performed to the finest level of detail possible (e.g., country, province, district, or city/town). In the case of a returning traveler report, we recorded the location visited as the site of the occurrence. The database was last updated on 4 October 2013.

The sources we used to create our initial database often described the occurrence of more than one DENV type at a time and/or spanned multiple years. Conversely, it was often the case that multiple sources were referring to the same outbreak. To account for these concerns we derived a standard definition of an occurrence as the following: one or more reports of confirmed infection(s) from a specific DENV type in a given unique location within a single year. Accordingly, if multiple reports confirmed the presence of a DENV type in the same location within the same calendar year, they were considered as a single occurrence record. It is thus important to stress that our definition of a type-specific occurrence does not relate to the actual number of cases reported (this information was not consistently available), but rather to the presence of that DENV type in a given area and year.

For mapping and descriptive purposes, province- or state-level administrative units (Admin1) served as our unique locations. When information was only available at the country (Admin0) level in a particular year, it was included in the database for those countries smaller in area than Queensland, Australia (the largest Admin1 unit in our database at 1.7 million km^2^). From this final Admin1-level occurrence database, a series of global maps were created for each DENV type across six time-periods between 1943 and 2013. We also present the number of DENV types ever reported in a given area across these time-periods. Graphs displaying yearly occurrences by world region and country were also created (Figures S1–S12 in the supplementary material online).

Although reports of suspected cases were excluded from the database, no restrictions were made regarding the specific diagnostic typing method used to confirm the DENV type(s) responsible for an occurrence because not all sources specified this information. This was particularly true for ProMED reports that often simply reported cases as confirmed without specifying the method of confirmation; however, for comprehensiveness in our database, the following identification method(s) were recorded: (i) virus isolation, (ii) PCR, (iii) plaque-reduction neutralization test (PRNT), (iv) not specified/other. The frequency of occurrences confirmed by each of these methods is described in Table S1 in the supplementary material online. In some cases, immunoglobulin M (IgM), immunoglobulin G (IgG), or hemagglutination inhibition (HI) assays were used to confirm infection with DENV, but used alone these methods are not useful for type-specific DENV determination owing to DENV type cross-reactivity [23]. As such, these were included in the not specified/other category.

## Mapping DENV spread

A total of 1 956 DENV1 occurrences, 1 931 DENV2 occurrences, 1 631 DENV3 occurrences, and 1 000 DENV4 occurrences were mapped at the Admin1 and small Admin0 level across the entire study period. An additional 1 811 confirmed DENV occurrences were not attributable to a specific type due to lack of testing and/or reporting.

**Figure 1:**
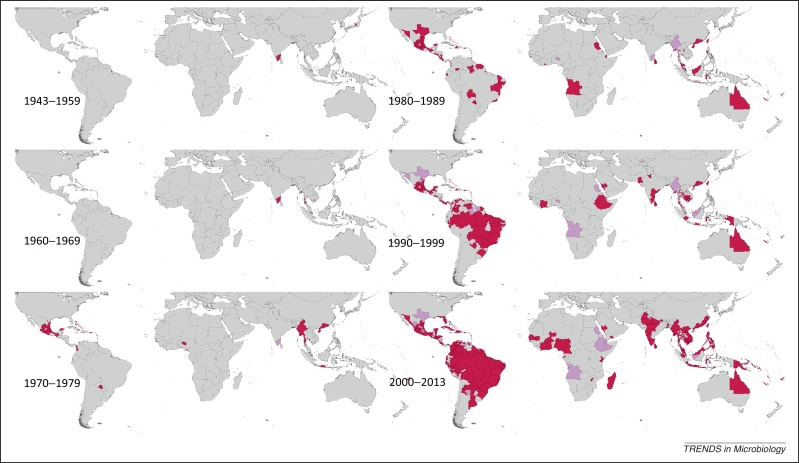
Spatial distribution of reported confirmed cases of DENV1 since 1943. Darker-colored areas represent cases that were confirmed in the given decade under consideration, whereas lighter-colored areas represent cases that had been previously reported but not in the current decade.

**Figure 2:**
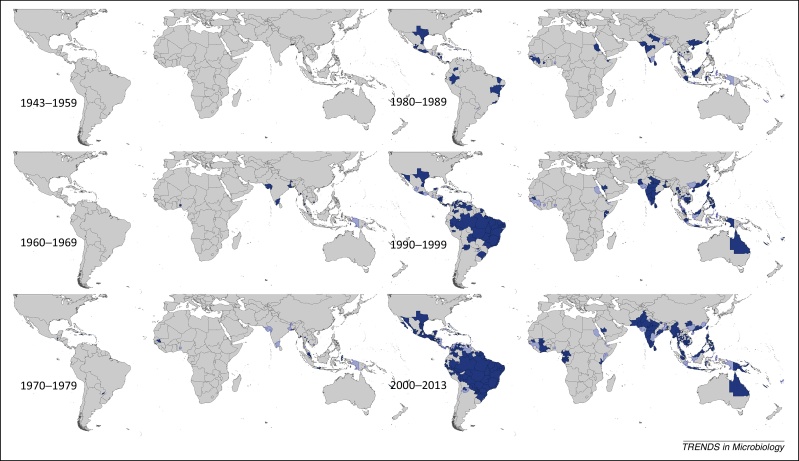
Spatial distribution of reported confirmed cases of DENV2 since 1943. Darker-colored areas represent cases that were confirmed in the given decade under consideration, whereas lighter-colored areas represent cases that had been previously reported but not in the current decade.

**Figure 3:**
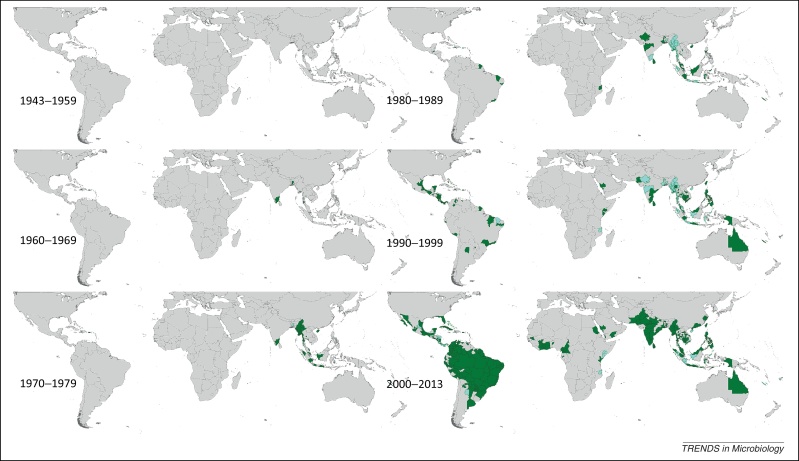
Spatial distribution of reported confirmed cases of DENV3 since 1943. Darker-colored areas represent cases that were confirmed in the given decade under consideration, whereas lighter-colored areas represent cases that had been previously reported but not in the current decade.

**Figure 4:**
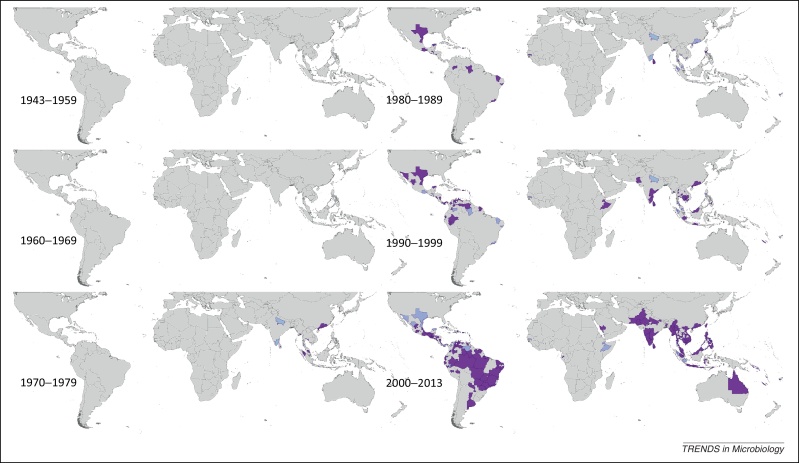
Spatial distribution of reported confirmed cases of DENV4 since 1943. Darker-colored areas represent cases that were confirmed in the given decade under consideration, whereas lighter-colored areas represent cases that had been previously reported but not in the current decade.

Our maps describe the reporting history of each DENV type for the periods 1943–1959, 1960–1969, 1970–1979, 1980–1989, 1990–1999, and 2000–2013 as presented in Figure 1, Figure 2, Figure 3, Figure 4.

These figures are complemented by additional graphs (Figures S1–S12 in the supplementary material online) that further break down the yearly distribution of occurrences for each DENV type by region (Africa, the Americas, and Asia) and by country within those regions. To simplify the number of categories presented in the supplementary material online, the Americas were considered to include North, Central, and South America, whereas Africa includes the African continent as well as Yemen and Saudi Arabia. The Asia region additionally includes Oceania and the Pacific Islands. The supplementary material also contains the summary of available information on type-specific diagnostic methods (Table S1 in the supplementary material online). Again, this is provided for descriptive purposes only because any occurrence described as confirmed was included in our database, regardless of diagnostic method, to obtain the most comprehensive picture of DENV type distributions.

### DENV1

Figure 1 displays the global spatial distribution of confirmed DENV1 occurrences by time period. DENV1 was first reported in 1943 in French Polynesia and Japan, followed by reports in Hawaii in 1944 and 1945. It was not until the late 1950s, however, that reporting of DENV1 in the Asian region constantly increased over time. It was first reported in Africa in 1984 in Sudan, and has been sporadically reported in the region ever since, with more continuous periods of reporting in Saudi Arabia between the mid-1990s and mid-2000s, as well as several years of reporting in Reunion in the mid-to-late 2000s. DENV1 was not reported in the Americas until 1977, when it was recorded in Barbados, Cuba, French Antilles, Grenada, Paraguay, and Puerto Rico. After these first recorded occurrences, reporting increased persistently across the region over the next few decades, with near-continuous reporting in Brazil, Mexico, and Puerto Rico in particular. Several other countries in the region began having more sustained reports in the 1980s and 1990s, including Colombia, Costa Rica, French Guiana, Paraguay, Peru, and Venezuela. Reporting of DENV1 peaked in 2005–2006, primarily attributable to recorded occurrences in the Americas. Since 1983, the Pan American Health Organization (PAHO), in collaboration with the US Centers for Disease Control (CDC) Dengue Branch in Puerto Rico, provided technical assistance for developing laboratory surveillance networks in several countries in the region, and this may partially explain increased reporting of all DENV types since this time.

### DENV2

DENV2 was first reported in 1944 in Papua New Guinea and Indonesia, followed by the Philippines in 1954 and 1956. Malaysia and Thailand have reported many consecutive years of DENV2 occurrence since the early 1960s, as well as Indonesia since the early 1970s and China, India, the Philippines, Sri Lanka, and Singapore since the 1980s. Continuous reporting did not occur in Cambodia and Vietnam until the 1990s. In Africa, DENV2 was reported in Nigeria multiple times between 1964 and 1968, but has not since been reported there. However, several sporadic occurrences have since been reported in the African region, with the most recent reports being from Gabon in 2010 and Kenya in 2013. DENV2 was reported in the Americas as early as 1953 in Trinidad and Tobago, but continuous reporting in the region did not begin until the late 1960s and early 1970s, most notably in Puerto Rico. Since this time, more and more Latin American countries have begun frequent reporting of DENV2, with continuous reporting in Brazil in particular since 1984 accounting for the majority of reporting of this type globally. In the 1990s, there was an increase in the number of the more severe hemorrhagic fever (DHF) cases in the Americas, possibly due to the replacement of the American DENV2 genotype with an imported and more virulent Asian one 24, 25, 26, 27. This increase in DHF cases may be responsible for the noticeable rise in DENV2 reporting in this region since that time, as seen in Figure 2. The largest number of occurrences reported to date was in 2005, with over 100 Admin1 and small Admin0 areas worldwide reporting DENV2 presence, primarily in the Americas.

### DENV3

DENV3 was first reported in 1953 in the Philippines and Thailand, and has been reported in Asia every year since 1962. Although many countries in Asia have reported DENV3 throughout the study period, Thailand most notably reported DENV3 every year between 1973 and 2010, with the most widespread reporting occurring between 1999 and 2002. Malaysia and Indonesia have also reported DENV3 frequently since the 1970s, as well as Sri Lanka since the early 1980s. Records of DENV3 in China, Vietnam, Cambodia, and Singapore have been fairly consistent since the mid-1990s. The first reports in the Americas were in Puerto Rico in 1963, which continued to report DENV3 until 1978, and then again from 1994 to 2008 owing to the introduction of a new DENV3 genotype from Asia [28]. The majority of other countries in the Americas did not start reporting the type until between the late 1980s and early 2000s. Particularly widespread reporting occurred in Brazil in the mid-2000s. In Africa, overall very little DENV3 has been reported since the first reports in 1984–1985 in Mozambique, and occurrence has mostly been sporadic, with the exception of more frequent reporting between 1994 and 2009 in Saudi Arabia.

### DENV4

DENV4 was reported first in 1953 in the Philippines and Thailand. Since this time the region has reported DENV4 yearly, most frequently in Thailand whose most widespread reporting occurred between 1999 and 2002. Sri Lanka has also reported DENV4 almost yearly since 1978. Although reporting by country has not been as consistent as for other DENV types, periods of more frequent reporting have occurred in the Indochina region as well as Indonesia, India, Myanmar, and French Polynesia. DENV4 was not reported in the Americas until 1981, when it was reported in Brazil, Cuba, Dominica, Puerto Rico, and the US Virgin Islands. Since this date, reporting has occurred yearly in the region, with particularly frequent reporting in Puerto Rico since the 1980s–1990s, Venezuela and Colombia since the 1990s, and Nicaragua, Brazil, and Peru since the late 1990s–mid 2000s.

### Co-circulation of DENV types

**Figure 5:**
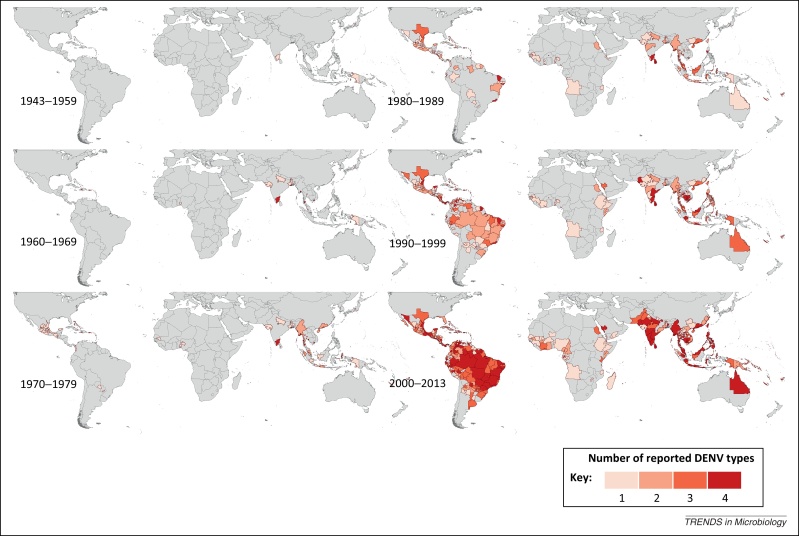
DENV Co-circulation. Cumulative number of DENV types reported by decade since 1943.

Figure 5 displays the cumulative number of DENV types having been reported in any given Admin1 and small Admin0 area by decade, and highlights the fact that, until the 1980s, the majority of areas had only reported one or two types of DENV. This figure allows the observation of potential increases in co-circulation of the four viruses, which may serve as a key indicator of progression toward hyperendemic transmission [29]. In the late 1980s, the number of types having been reported within a single area began to increase as more cost-effective and less labor-intensive type-specific diagnostic tests (e.g., PCR) were developed for dengue [30]. This may also explain increases in global reporting of specific DENV types at this time, although the number of reported DENV types has since continued to increase in many areas across Latin America and the Caribbean islands, as well as in Southeast Asia, the Indian subcontinent, Indonesia, and Australia. This is particularly noticeable in the map representing 2000–2013, by which time the majority of Brazilian provinces as well as much of Mexico, India, and Indonesia had reported every DENV type. Although few areas in Africa have reported all four DENV types, by the 2000s several areas had reported three. However, it is unclear whether these all represent the persistently transmitted epidemic form of the virus, or are only sporadic overspills from the sylvatic transmission cycle.

## Exponential growth in DENV type reporting

Reporting of DENV type is irregular and affected by many types of bias, in particular in locations with less virological diagnostic capacity, and thus our database represents an opportunistic sample of occurrence. As such, it is important to remember that an absence of reporting for one DENV type is not synonymous with an absence of its occurrence. It is also important to remember that our definition of a type occurrence does not relate to the actual number of cases reported, but rather to the presence of the DENV type in a given area and year. Furthermore, because our definition of a type occurrence does not relate to the actual number of cases reported, but rather the presence of the DENV type in a given area and year, it is impossible to make inferences about the relative prevalence of each type in any given location. That said, the records that support this review comprise the most comprehensive type-specific DENV database to date, and its breadth allows a much more detailed depiction of spread than was previously available.

Although there is general agreement that several factors have led to an expansion of the geographic range of dengue over our study period, and particularly in the latter half of the 20th century 6, 31, 32, 33, 34, 35, 36, the variability in reporting practices over time as well as by region and country must be considered alongside apparent patterns of expansion [37]. Improvements in reporting capacity over time will have had a significant effect on the number of cases being reported, compounded by the fact that increasing co-circulation of DENV types may be associated with more severe disease outcomes which are therefore more likely to be reported. However, this study makes it clear that dengue detection has increased dramatically across the globe since 1943, with some DENV types being newly reported in particular areas more rapidly than others. DENV1 was reported to occur the most times during this 70 year study period, followed by DENV2, DENV3, and DENV4. The overall number of confirmed type-specific DENV events particularly escalated in the 1990s, primarily comprising increases in DENV1 and DENV2 detection in the Americas owing to the availability of rapid diagnostic tests. In Asia, although DENV2 reporting has increased rapidly, DENV3 reporting has surpassed that of DENV1. The greatest increase in reporting of DENV3 occurred in the 1990s, predominantly in the Americas. DENV4 reporting spread the least rapidly during the 70 year period, although it has been consistently reported in ever-greater numbers since the 1980s, particularly in Asia and the Americas.

Although documenting dramatic increases in DENV reporting across Asia and the Americas, our review also underscores the fact that there still remains a dearth of type-specific DENV information in many parts of Africa – where our understanding of the evolutionary history and current dynamic of DENV transmission and spread is also poorest. Both research efforts and numbers of reports have remained relatively low here compared to Asia and the Americas, although several arboviral studies have been conducted in the region since the 1980s 38, 39, 40, 41, 42, 43, 44, 45, 46, 47, 48, 49, 50, 51, 52, 53, 54, 55, 56. This problem is made worse by the lack of DENV type-specific information in the few countries where occurrence reports are available. This is particularly true in the countries of east Africa where evidence for dengue presence is high [21], but little information exists about the specific location(s) within these countries where DENV has occurred or about the DENV type(s) responsible for occurrence.

## Research outlook

Additional research questions can be explored with use of the database presented here, such as those surrounding the importance of travel, migration, and commercial trade in the spread of dengue, the introduction of novel DENV types (and/or genotypes) into locations where DENV is or is not already present [57], and DENV population structure and evolution, as well as casting light on how human immunity mediates DENV transmission at the micro and macro scales (Box 1) [58]. The ability to answer these questions will complement phylogenetic studies, and may even be integrated with phylogeographic studies of DENV evolution and dispersal on a regional and global scale 59, 60, 61. The comprehensive database that has been compiled will be made publicly available (via http://figshare.com [62]) such that it can be directly referred to in order to facilitate DENV type identification in historic samples, thereby providing information about what specific viruses were circulating in an area at a particular time. Tracking the spread of DENV types also has important implications for ongoing research [15] analyzing changes in dengue endemicity in a given area over time.

Box 1 **Outstanding questions**

• How can we quantify the importance of travel, migration, and commercial trade in the global spread and evolution of DENV types?
• Does co-circulation of DENV types increase the incidence of more severe disease outcomes in a given area? A greater number of seroprevalence studies involving active surveillance are required to answer this question.
• What is the distribution of DENV types in Africa? Although returning traveler reports are useful for establishing the presence of certain types in some African countries, these reports are currently infrequent and sporadic. Greater surveillance efforts are needed in Africa.

## Continuing spread has global implications

Although international transmission of disease is not new, it is notable that airline passenger numbers have increased by 9% annually since 1960, enabling infected human hosts to move the viruses long distances more quickly 27, 63. Increased urbanization along with substandard housing, unreliable water supply, and poor sanitation provide an environment for *Ae. aegypti* proliferation in close proximity to human hosts. In the Americas, this may have been exacerbated by the collapse of the *Ae. aegypti* eradication program in the 1970s [8]. Increases in the size of resource-poor urban populations in Latin America and the Caribbean in the late 1980s and early 1990s may have supported the establishment and spread of all four DENV types in the Americas [64]. According to the United Nations Population Division, between 2000 and 2030, Africa and Asia together are expected to account for four-fifths of all urban growth in the world (see http://esa.un.org/unpd/wpp/Documentation/pdf/WPP2012_Volume-I_Comprehensive-Tables.pdf). Therefore, it is essential that we closely monitor the ongoing and future spread of DENV types in these regions to understand, detect, and respond better to the global burden of dengue disease.

Finally, because our maps have underscored the ability of all four DENV types to expand into new territories, the crucial need for a DENV vaccine which protects against all four known types is made ever more apparent [64]. It is also important to note the recent suggestion of a fifth DENV type [65] which, if found to be as transmissible as the other four DENV types, might follow a similar pattern of geographical spread as seen with these types over the past two decades. If sustained transmission of a fifth type did occur in human populations, this would be an additional consideration for vaccine development efforts 64, 66, and an up-to-date map of the spread of DENV types would be essential.

## Concluding remarks

In sum, this review offers the most contemporary understanding of DENV type-specific geographic distributions from 1943 to 2013, providing a starting point and rationale for charting the ongoing global spread of each DENV type. Specifically, the increasing co-circulation of types in most regions of the world – particularly in Latin America and Asia – has important implications for patterns in disease severity and hyperendemicity, as well as for ongoing vaccine efforts. It also highlights a paucity of DENV type-specific geographic information in many locations across the globe that needs to be urgently addressed.

## Glossary

Flavivirus: a family of single-stranded RNA viruses transmitted primarily by ticks and mosquitoes.
Medical Subject Headings (MeSH) terminology: a wide-ranging and regulated vocabulary for the purpose of indexing and searching for journal articles and books in the life sciences; created by the US National Library of Medicine.
Sylvatic: denoting certain diseases contracted by and affecting wild animals, and the pathogens causing them.
