# Supplementary material for: Global spread of dengue virus types: mapping the 70 year history
Source: Trends Microbiol. 2014 Mar;22(3):138–46. doi: 10.1016/j.tim.2013.12.011 (PMC3946041; doi:10.1016/j.tim.2013.12.011)
Supplement: Supplementary file 1 [file mmc1.docx]

Supplementary Material

Global spread of dengue virus types: mapping the 70-year history

**Jane P. Messina^1^, Oliver J. Brady^1^, Thomas W. Scott^2,3^, Chenting Zou^1^, David M. Pigott^1^, Kirsten Duda^1^, Samir Bhatt^1^, Leah Katzelnick^4^, Rosalind E. Howes^1^, Katherine E. Battle^1^, Cameron P. Simmons^5,6,7^, and Simon I. Hay^1,3^**

^1^Spatial Ecology and Epidemiology Group, Department of Zoology, University of Oxford, South Parks Road, Oxford OX1 3PS, United Kingdom.

^2^Department of Entomology, University of California Davis, Davis, California 95616, United States of America.

^3^Fogarty International Center, National Institutes of Health, Bethesda, Maryland 20892, United States of America.

^4^Department of Zoology, University of Cambridge, Cambridge, CB2 3EJ, United Kingdom.

^5^Oxford University Clinical Research Unit, Hospital for Tropical Diseases, Ho Chi Minh City, Vietnam.

^6^Centre for Tropical Medicine, University of Oxford, Churchill Hospital, Oxford OX3 7LJ, United Kingdom.

^7^Nossal Institute of Global Health, University of Melbourne, Parkville, Victoria, Australia

Corresponding author: Jane P. Messina ([jane.messina@zoo.ox.ac.uk](mailto:jane.messina@zoo.ox.ac.uk))

**Table S1. Diagnostic and typing methods recorded in our database^a^**

|  | **DENV1** | **DENV2** | **DENV3** | **DENV4** | **Any confirmed**  **DENV type** |
| --- | --- | --- | --- | --- | --- |
| **Typing method** | *%* | *%* | *%* | *%* | *%* |
| **Virus isolation** | 42.6 | 46.5 | 44.8 | 44.3 | 38.2 |
| **PCR** | 33.9 | 38 | 40.5 | 40.2 | 32.5 |
| **PRNT** | 5.7 | 7.5 | 6.7 | 8.5 | 5.2 |
| **Not specified / other** | 46.3 | 39.5 | 41.5 | 42.1 | 49.1 |

^a^Multiple DENV types were often identified with a single method, and multiple methods were often employed to identify a single DENV type.

**
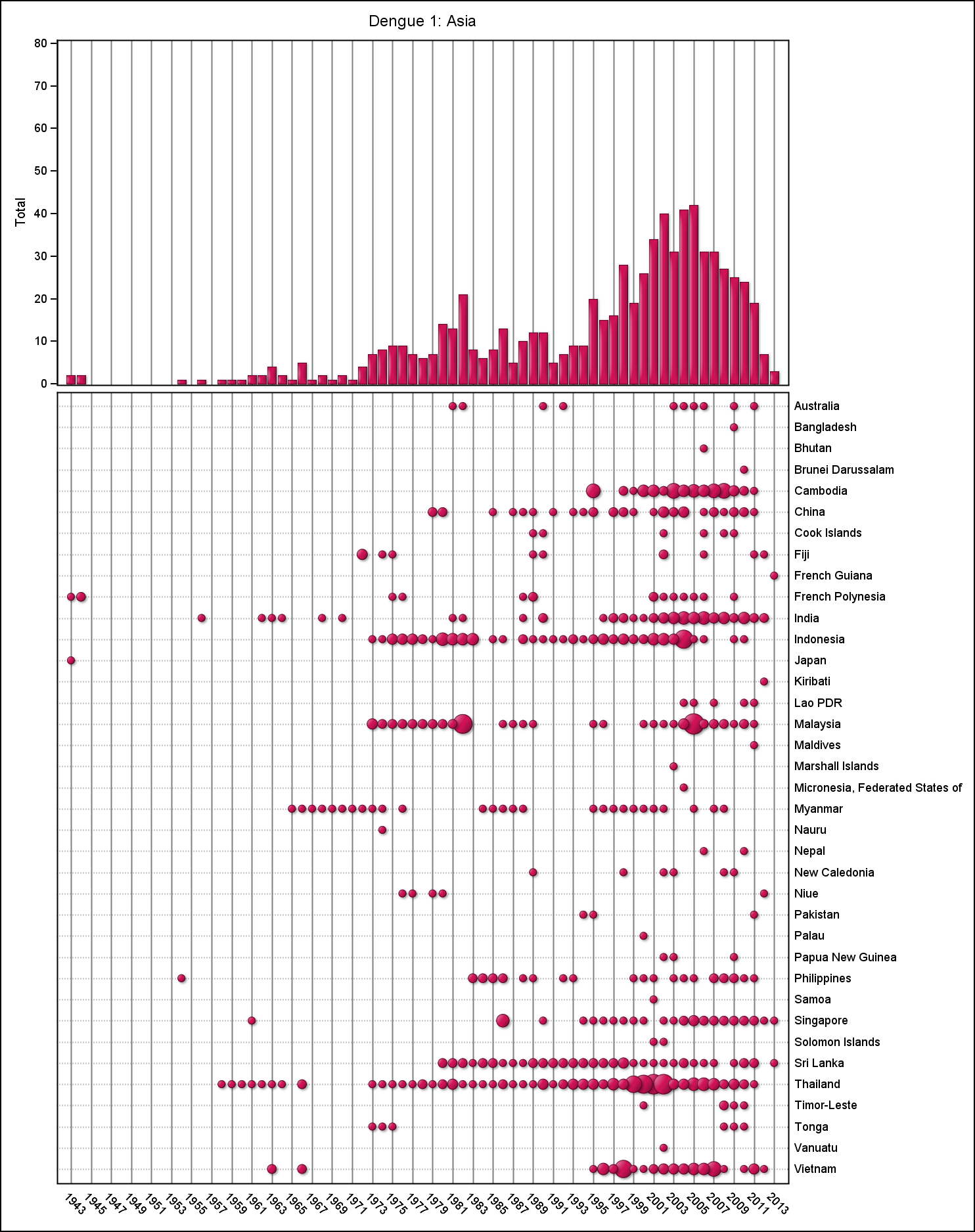
**

Figure S1. Yearly distribution of reported confirmed cases of DENV1 since 1943 in Asia. The histogram displays the totals for the entire region, with bubble plots showing positive occurrence in Admin1 (province-level) and small Admin0 (country-level) areas within individual countries. The size of the circles represents the counts within each country.


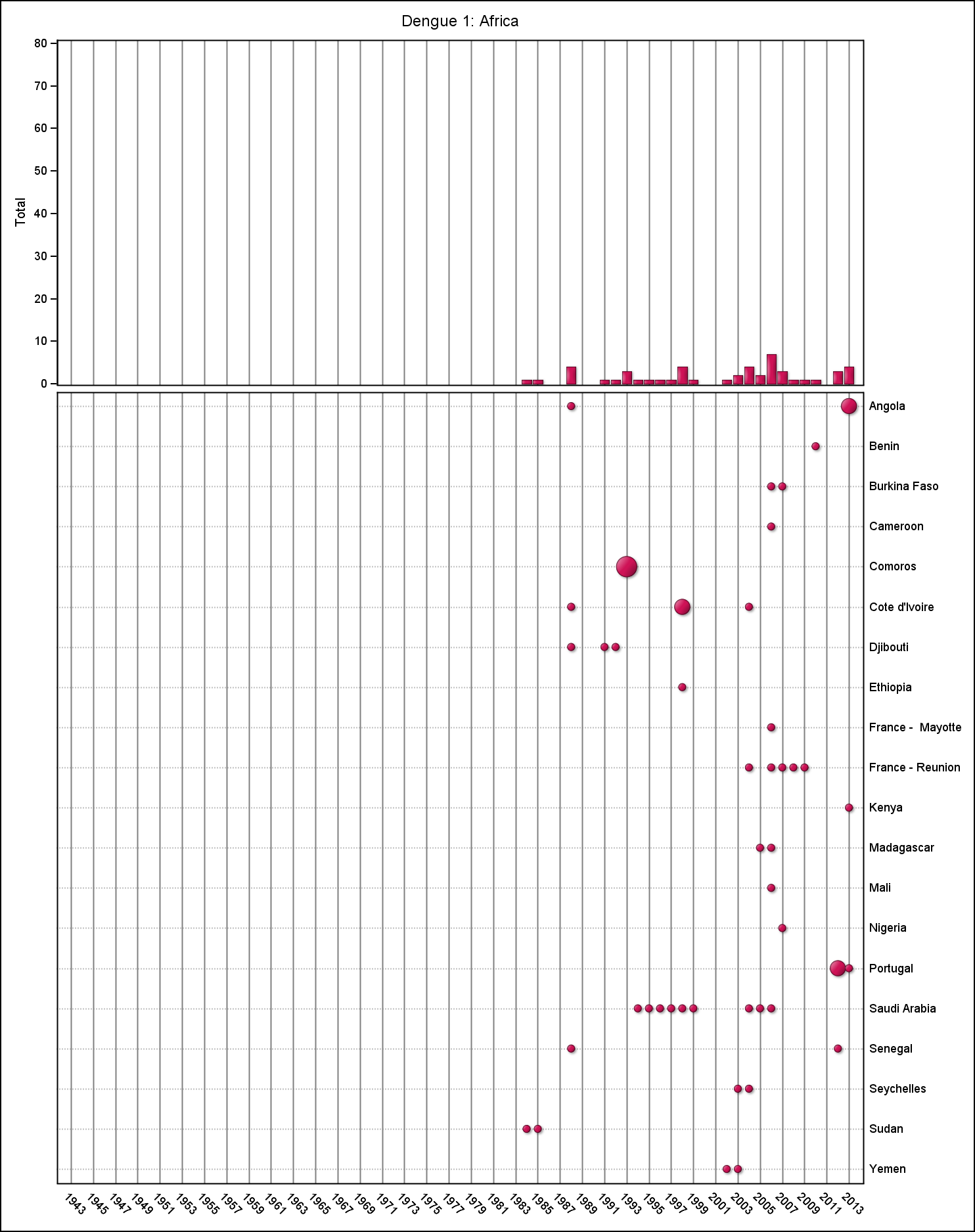


Figure S2. Yearly distribution of reported confirmed cases of DENV1 since 1943 in Africa. The histogram displays the totals for the entire region, with bubble plots showing positive occurrence in Admin1 (province-level) and small Admin0 (country-level) areas within individual countries. The size of the circles represents the counts within each country.


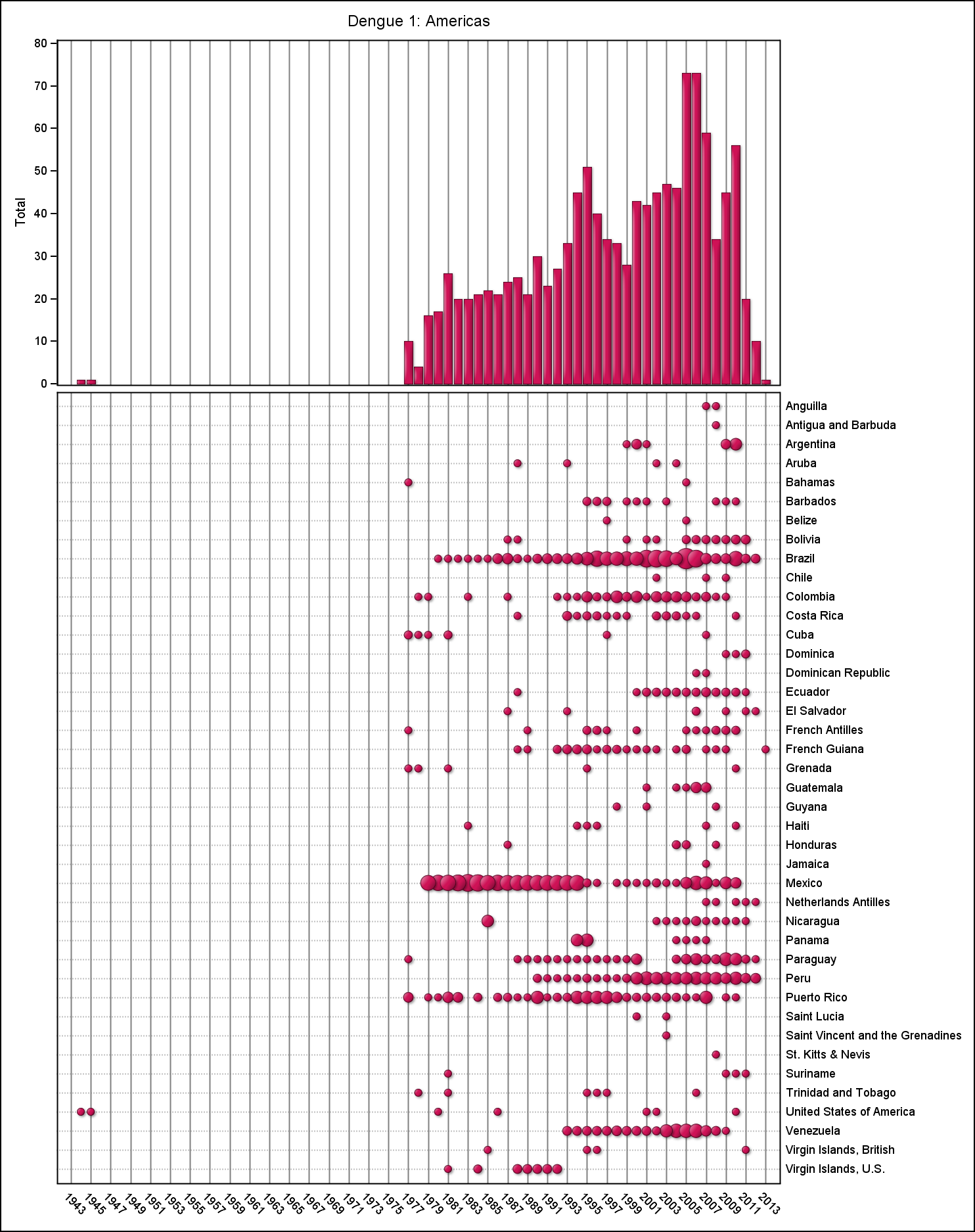


Figure S3. Yearly distribution of reported confirmed cases of DENV1 since 1943 in the Americas. The histogram displays the totals for the entire region, with bubble plots showing positive occurrence in Admin1 (province-level) and small Admin0 (country-level) areas within individual countries. The size of the circles represents the counts within each country.


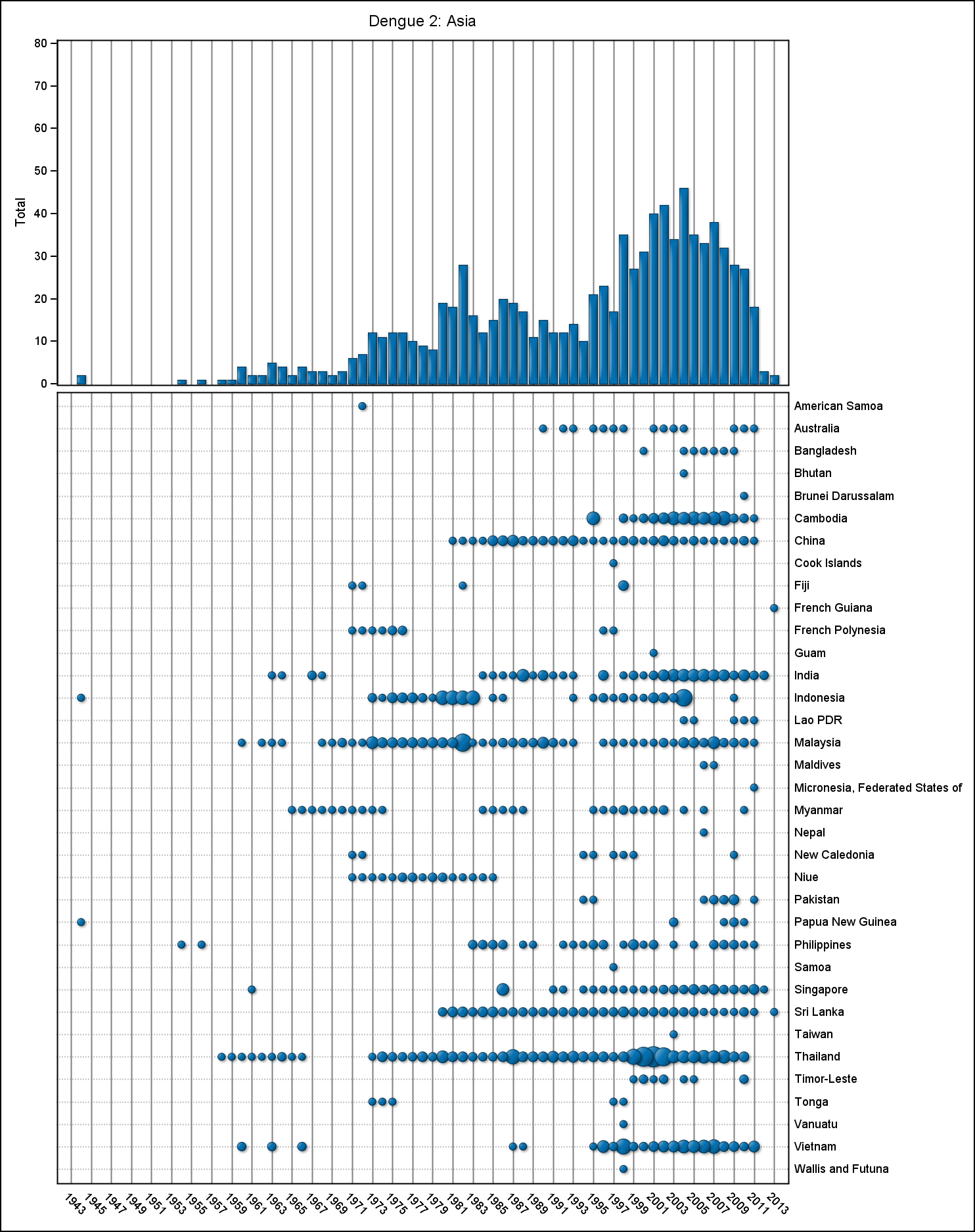


Figure S4. Yearly distribution of reported confirmed cases of DENV2 since 1943 in Asia. The histogram displays the totals for the entire region, with bubble plots showing positive occurrence in Admin1 (province-level) and small Admin0 (country-level) areas within individual countries. The size of the circles represents the counts within each country.


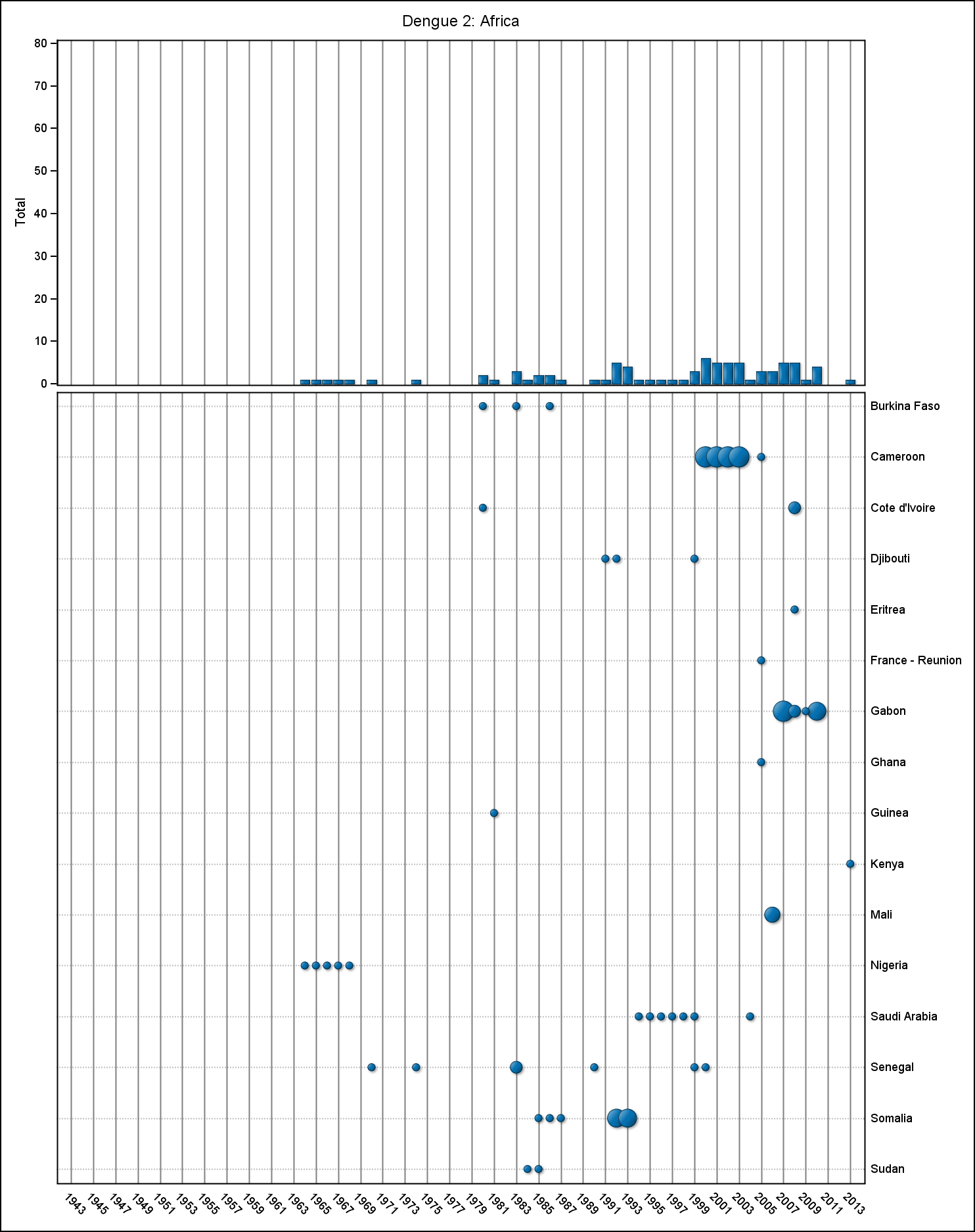


Figure S5. Yearly distribution of reported confirmed cases of DENV2 since 1943 in Africa. The histogram displays the totals for the entire region, with bubble plots showing positive occurrence in Admin1 (province-level) and small Admin0 (country-level) areas within individual countries. The size of the circles represents the counts within each country.


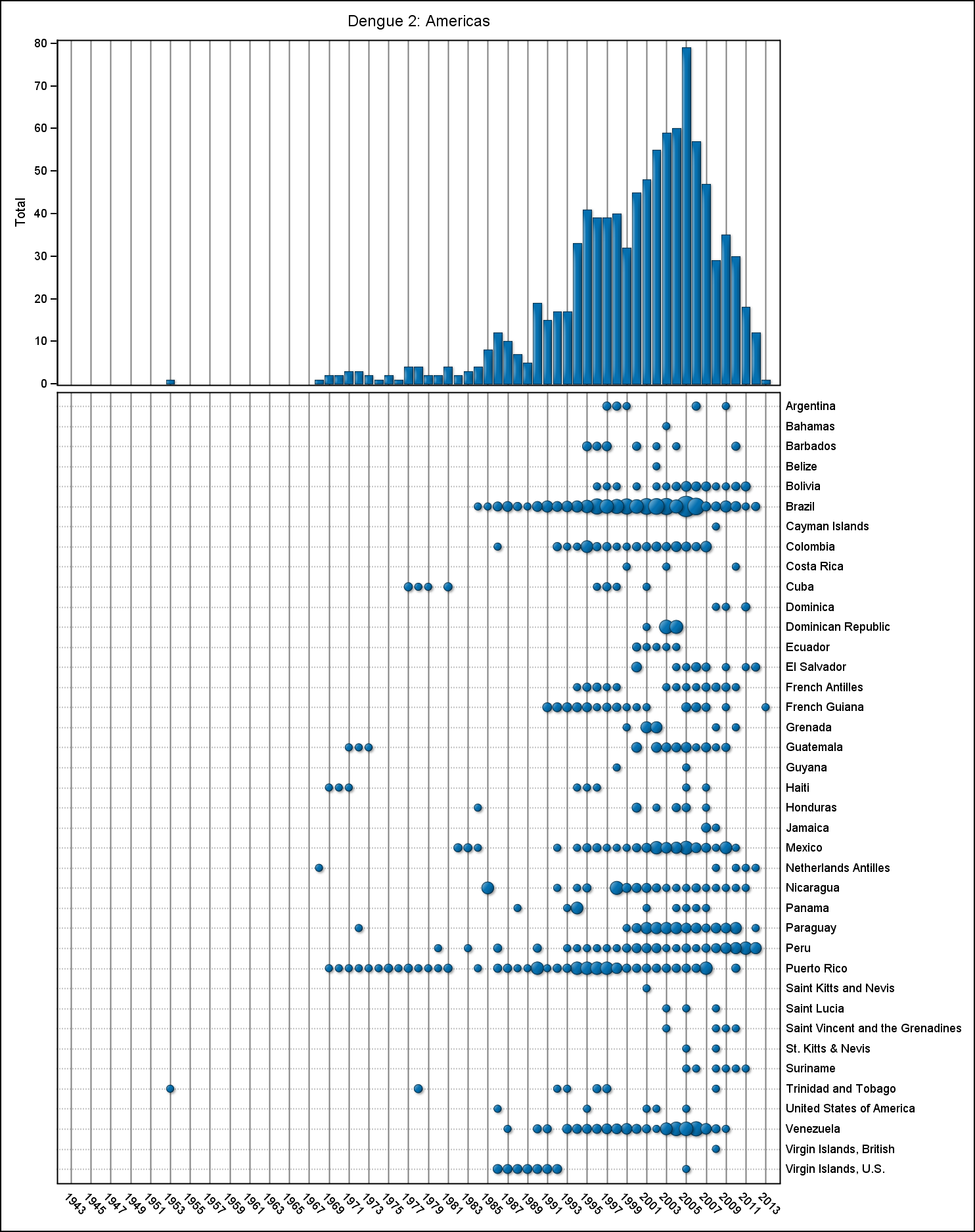


Figure S6. Yearly distribution of reported confirmed cases of DENV2 since 1943 in the Americas. The histogram displays the totals for the entire region, with bubble plots showing positive occurrence in Admin1 (province-level) and small Admin0 (country-level) areas within individual countries. The size of the circles represents the counts within each country.


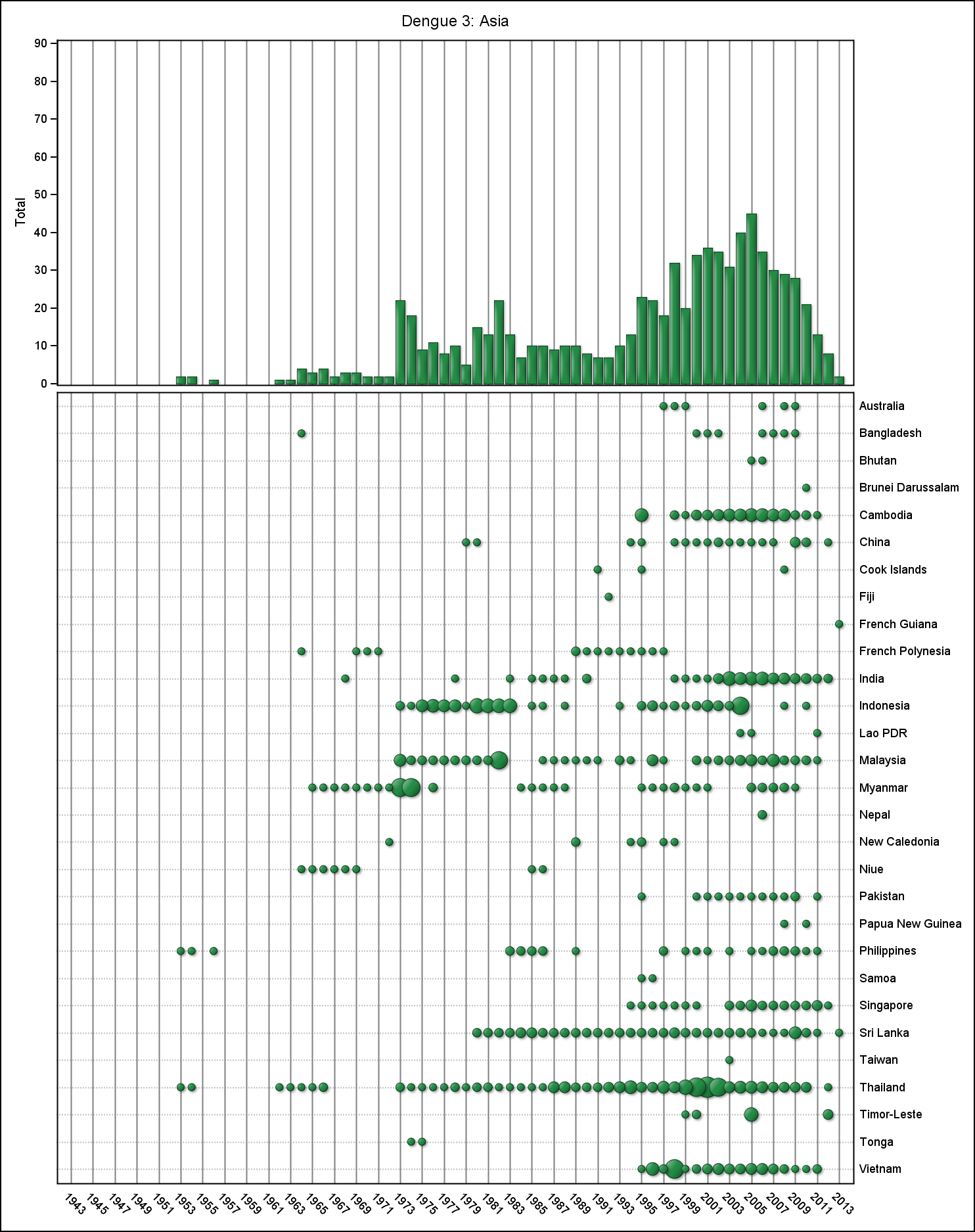


Figure S7. Yearly distribution of reported confirmed cases of DENV3 since 1943 in Asia. The histogram displays the totals for the entire region, with bubble plots showing positive occurrence in Admin1 (province-level) and small Admin0 (country-level) areas within individual countries. The size of the circles represents the counts within each country.


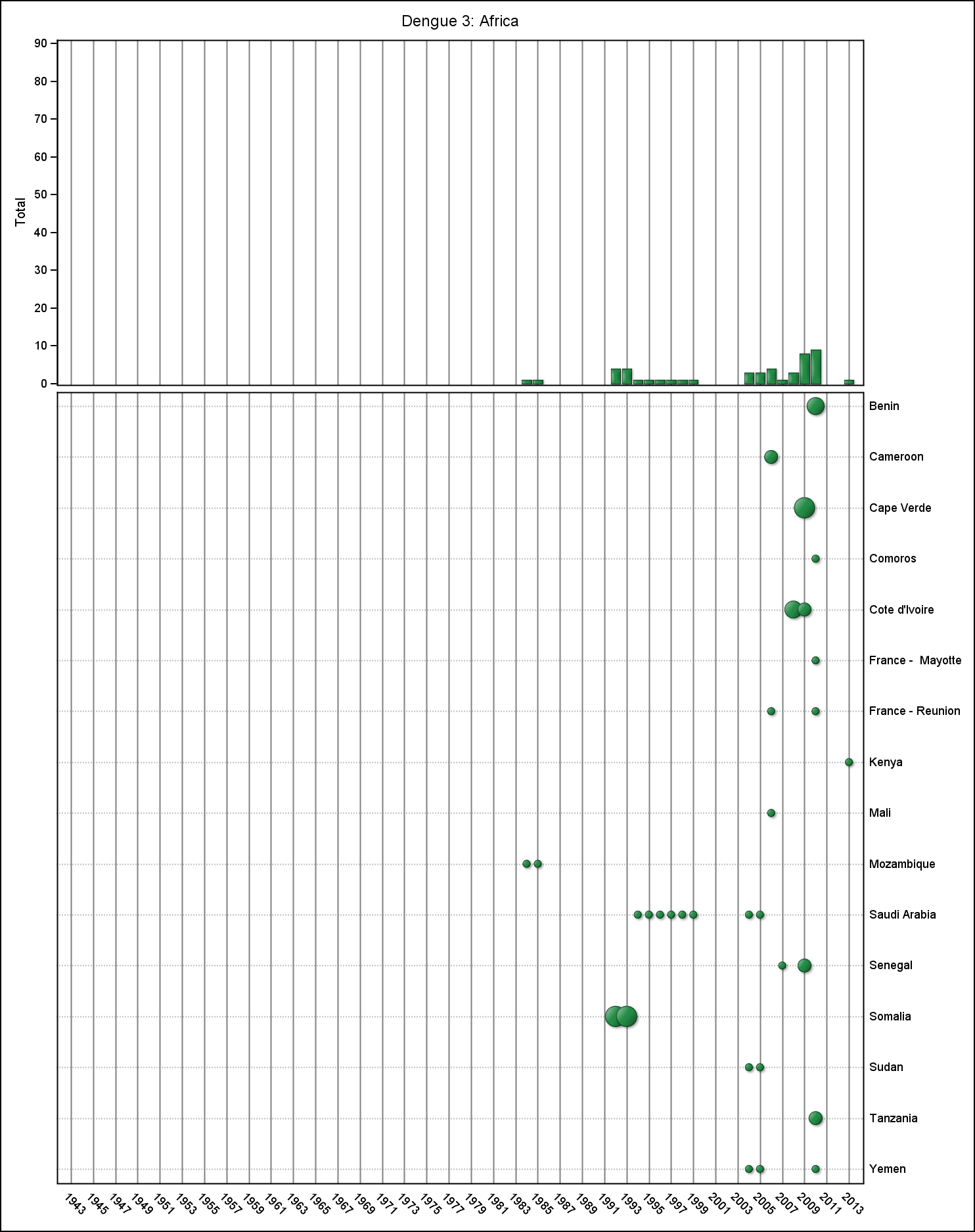


Figure S8. Yearly distribution of reported confirmed cases of DENV3 since 1943 in Africa. The histogram displays the totals for the entire region, with bubble plots showing positive occurrence in Admin1 (province-level) and small Admin0 (country-level) areas within individual countries. The size of the circles represents the counts within each country.


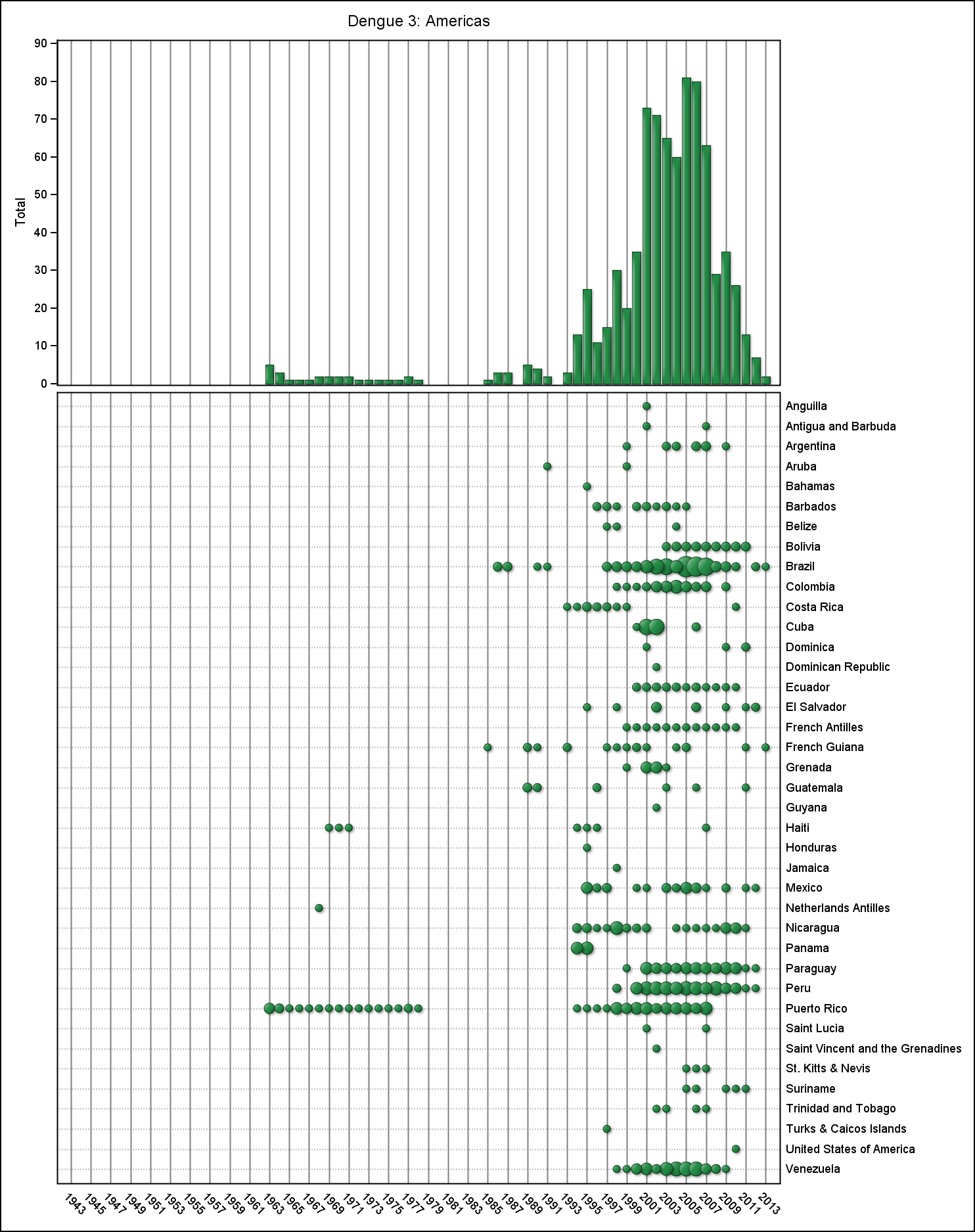


Figure S9. Yearly distribution of reported confirmed cases of DENV3 since 1943 in the Americas. The histogram displays the totals for the entire region, with bubble plots showing positive occurrence in Admin1 (province-level) and small Admin0 (country-level) areas within individual countries. The size of the circles represents the counts within each country.


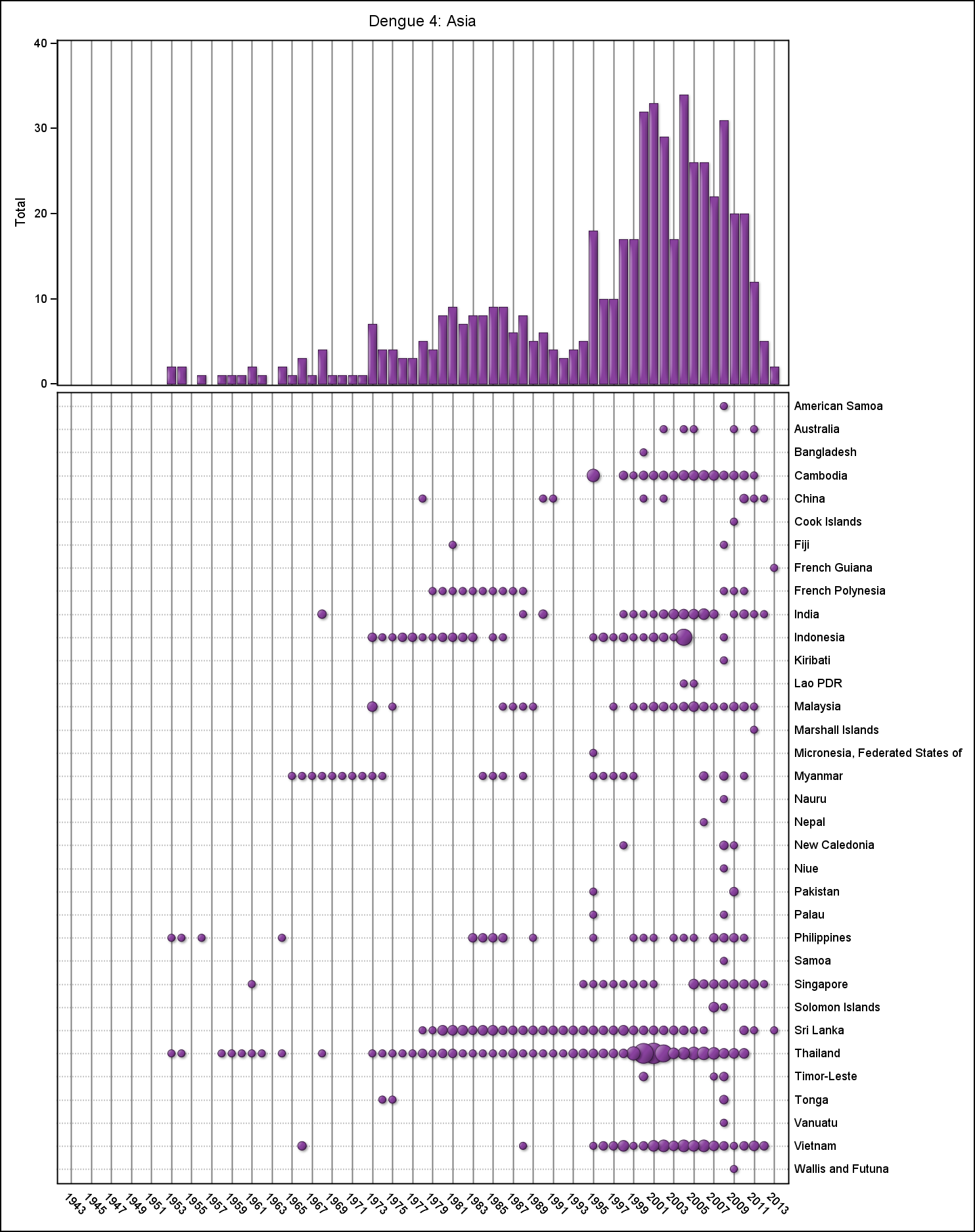


Figure S10. Yearly distribution of reported confirmed cases of DENV4 since 1943 in Asia. The histogram displays the totals for the entire region, with bubble plots showing positive occurrence in Admin1 (province-level) and small Admin0 (country-level) areas within individual countries. The size of the circles represents the counts within each country.


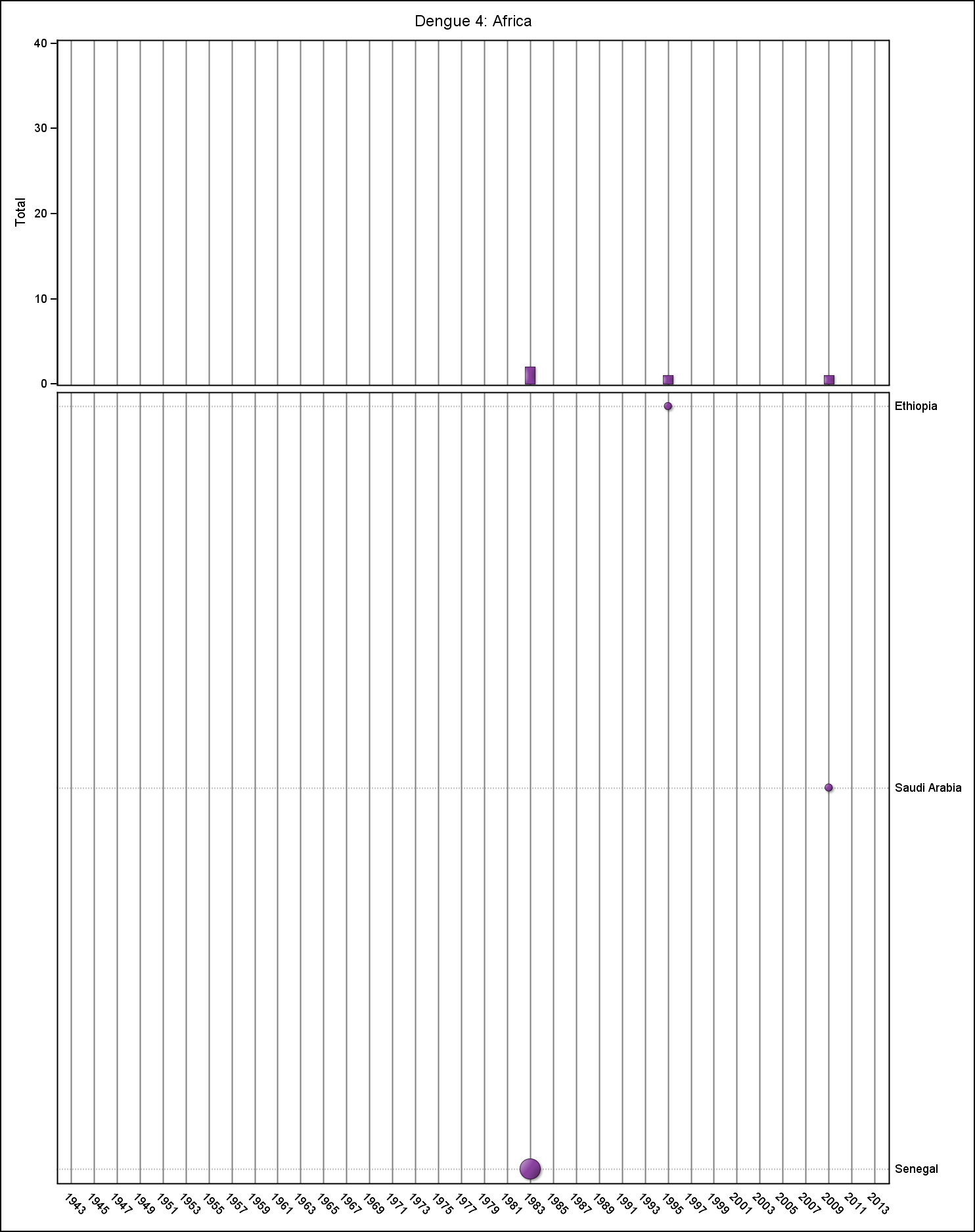


Figure S11. Yearly distribution of reported confirmed cases of DENV4 since 1943 in Africa. The histogram displays the totals for the entire region, with bubble plots showing positive occurrence in Admin1 (province-level) and small Admin0 (country-level) areas within individual countries. The size of the circles represents the counts within each country.


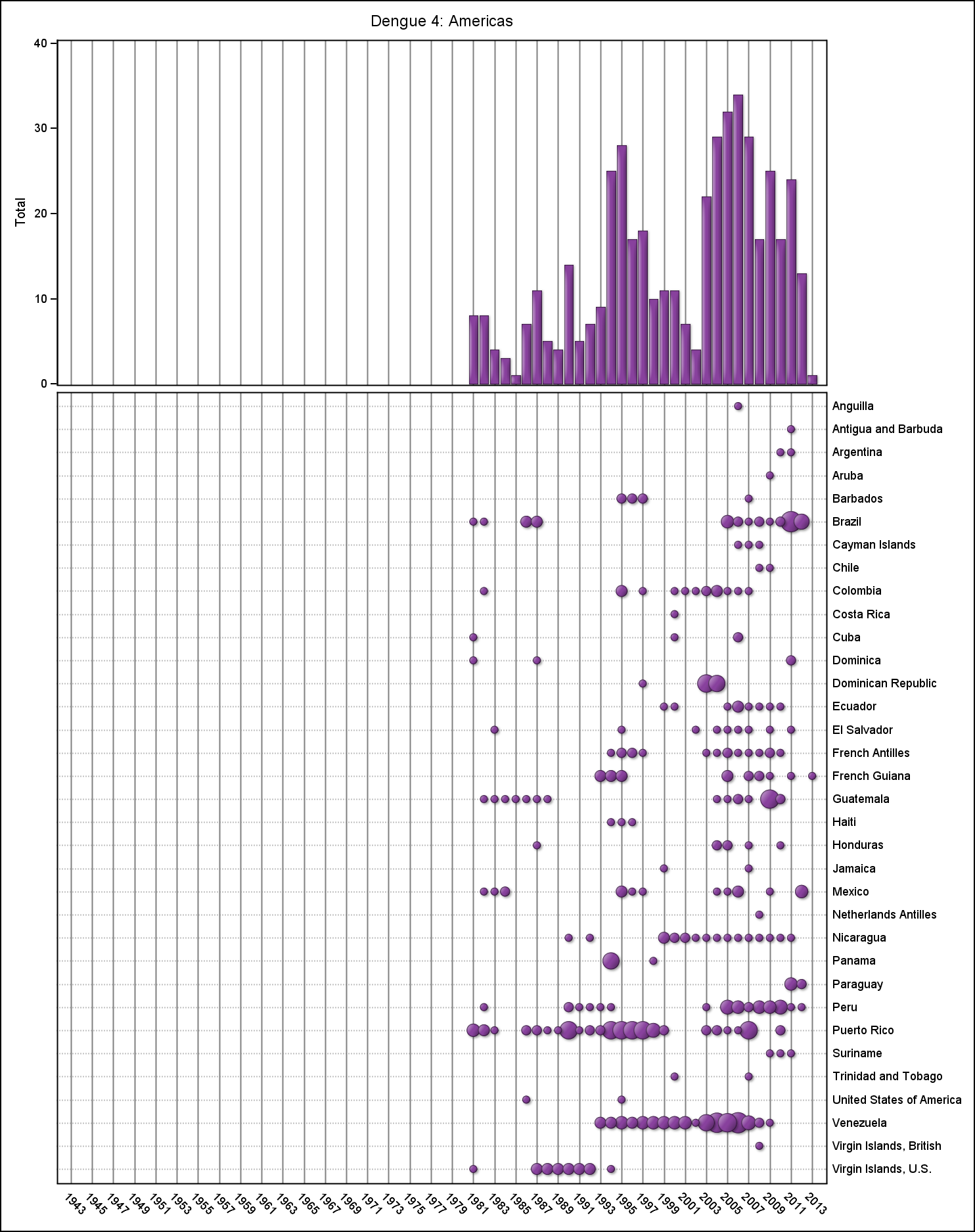


Figure S12. Yearly distribution of reported confirmed cases of DENV4 since 1943 in the Americas. The histogram displays the totals for the entire region, with bubble plots showing positive occurrence in Admin1 (province-level) and small Admin0 (country-level) areas within individual countries. The size of the circles represents the counts within each country.
